# A Rare Case of Split Notochord Syndrome

**DOI:** 10.21699/jns.v6i1.487

**Published:** 2017-01-01

**Authors:** Sudhir Singh, VP Bothara, JD Rawat, Digamber Chaubey, Piyush Kumar, Gurmeet Singh

**Affiliations:** Department of Paediatric Surgery, King George’s Medical University, Lucknow, India

**Dear Sir**

We treated a one day old baby presenting with an extensive lumbosacral deformity, hydrocephalus and apparent enteric segments in the dorsal midline with abnormal genitalia, accompanied by an enteric fistula and imperforated anus. The malformation fits into split notochord syndrome. The baby died as a result of sepsis before surgical treatment could be attempted. Patient was full term caesarian delivery of twin pregnancy. The other baby was a normal live male. There was no history of exposure to teratogenic agents or family history of congenital defects. On admission, the patient was cold, lethargic and tachycardiac. Baby had en-large head with bulging fontanelle, dorsal midline spinal defect with prolapsing bowel loops (Fig.1). Anal opening was not present and baby had abnor-mal genitalia, which was extensively shifted dorsally with bifid scrotum, peno-scrotal transposition with right gonad not palpable. There was no leakage of cerebrospinal fluid or visible exposure of neural tissue elements. He could move his lower limbs. Neither urinary retention or dribbling nor a palpable bladder was present. The cranial ultrasound showed type II Arnold Chiari malformation, hydrocephalus, and cerebral cortex abnormal development. The baby was expire on day 3 of life before planning further investigations and surgical interventions due to sepsis.

The split notochord syndrome was first described by Rembe in 1887. [1] This syndrome contains, vertebral anomalies, central nervous system abnormalities, and intestinal anomalies. In this syndrome as a cleft in the dorsal midline of the body through which intestinal segments are exteriorized. Central nervous system abnormalities are always present. These babies may present with functional spinal cord defects. The embryological origin of this anomaly is still unknown. The oldest theory suggests the persistence of a primitive neurenteric canal connecting the amniotic cavity to the dorsum of the embryo in the third week of gestation. [2] Another theory attribute this discrepancy to the varied positions of Hensen’s node (accessory neurenteric canal). [3] A recent theory suggests a primary notochord defect resulting in secondary changes to the paraxial mesoderm, which is responsible for the formation of the spinal column, giving rise to a medial interosseous space. Through this space, the endoderm and the underlying primitive intestine herniate, adhere to the dorsal ectoderm, and eventually rupture. [4] A poor prognosis for survival has been described in the literature, with only few survivors being reported.

**Figure:**
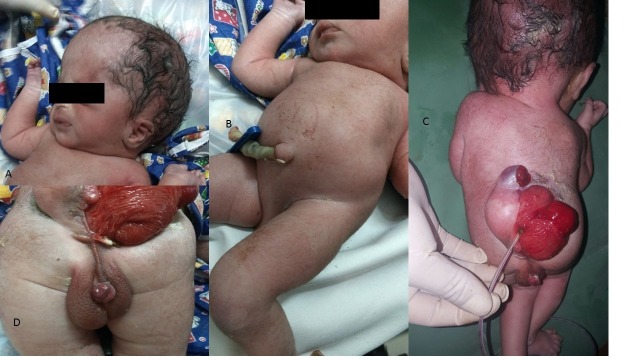
Figure 1-A Macrocephaly, B, D dorsally shifted abnormal genitalia, C Prolapsed bowel at spinal lesion with enteric fistula.

## Footnotes

**Source of Support:** Nil

**Conflict of Interest:** None

## References

[1] Kgur FM, Ozdemir T, Olguner M, Erbayrektar S, Ozer E, Aktug T. A case of split notochord syndrome: presence of dorsal enteric to the dorsal enteric fistula. J Pediatr Surg. 1998; 33:1317-9. 10.1016/s0022-3468(98)90179-8 9722015

[2] Bremer JL. Dorsal intestinal fistula, accessory neurenteric canal, dyastematomielia. Arch Pathol. 1952; 54:132-8. 14943345

[3] Rauzzino MJ, Tubbs RS, Alexander E III, Grabb PA, Oakes WJ. Spinal neuroenteric cysts and their relation to more common aspects of occult dysrafism. Neurosurg Focus [serial on the Internet] 2001 [cited 2004 Jan 19]; 10: 10. Available from: http://www.neurosurgery.org/focus. 10.3171/foc.2001.10.1.3 16749754

[4] Jesus LE, França CG, A rare variant of neuroenteric cyst: split notochord syndrome. J Pediatria. 2004; 80:77-80. 14978554

